# Non-traumatic scapholunate dissociation in a 10-year-old female: A case report

**DOI:** 10.3389/fsurg.2022.917555

**Published:** 2022-09-01

**Authors:** Diletta Bandinelli, Alessia Pagnotta, Edoardo Maria Pieracci, Luca Basiglini, Angelo Gabriele Aulisa

**Affiliations:** ^1^ Department of Orthopaedics, Bambino Gesù Children's Hospital (IRCCS), Rome, Italy; ^2^ Hand and Microsurgery Unit, Jewish Hospital, Rome, Italy; ^3^ Department of Orthopaedics and Traumatology, San Paolo Hospital, Civitavecchia, Italy; ^4^ University of Cassino and Southern Lazio, Cassino, Italy

**Keywords:** scapholunate dissociation, child, young age, carpal injuries, non-traumatic

## Abstract

**Introduction:**

Severe or minor repetitive trauma, inflammation, infection, tumors, and congenital ligamentous laxity have been etiologically implicated in scapholunate dissociation (SLD). While a few cases of patients with asymptomatic SLD have been reported in the literature, despite radiographically demonstrated widened scapholunate angles and rotatory subluxation of the scaphoid bone, these patients experienced only mild or no pain and no dorsal intercalated segment instability deformity. Here, we report the case of a monolateral non-traumatic SLD in a young 10-year-old girl that led to an important range of motion impairment with no wrist pain. The case represents a rarity for no previous history of trauma, young age, and no pain.

**Main symptoms and important clinical findings:**

In our patient, an examination revealed a reduced range of motion in the left wrist and no pain during passive or active mobilization. The X-ray showed a 16 mm scapholunate gap in the anteroposterior roentgenogram. In this case, we suggested that congenital or developmental ligamentous laxity may be the cause of SLD. The diagnostic assessment was completed with a wrist MRI and CT.

**Therapeutic interventions and outcomes:**

The patient underwent an open dorsal surgery: we directly reduced the dislocated bones and fixed them with five percutaneous 1 mm k-wires. Finally, the scapholunate ligaments were repaired using bone-absorbable anchor sutures. The wrist was immobilized in a volar cast for 8 weeks. The patient was able to resume her daily life activities (included sport) within 12 months.

**Conclusion:**

Carpus injuries are rare in children, and treatment, especially for young-age patients, is fraught with risks and remains controversial. Our case demonstrates that the patient has had a good clinical outcome. The physio-rehabilitation program for this patient has been of long duration. Most previous studies have shown excellent clinical results after an average of 2.4 years.

## Introduction

Scapholunate dissociation (SLD) is a consequence of a lesion of the ligamentous complex holding two carpal bones, the scaphoid and lunate. The scapholunate ligament is made of three parts: volar, intermediate, and dorsal, which is the strongest component. In particular, when the whole ligament or the dorsal breaks, a radiocarpal joint instability occurs. SLD occurrence has been consistently attributed to previous trauma that typically occurs with a hyperextended and an ulnar-deviated wrist during a fall. Approximately 5% of SLD is associated with distal radius fractures, in particular fractures of the radial styloid. Even if the main cause of SLD is trauma, other etiologies have been reported in the literature, such as rheumatoid arthritis, spastic paresis, and congenital ligament laxity (1).

SLD has been noted to occur in the setting of severe or minor repetitive trauma, infection, tumors, inflammation, infection, and congenital ligamentous laxity. SD ligament rupture is the first step of a hand lesion series: carpal instability leads to a volar rotation of the scaphoid and dorsal rotation of the lunate, resulting in a misalignment and later arthrosis between the carpal bones and the radius (2).

While a few cases of patients with asymptomatic SLD have been reported, despite a radiographically demonstrated scapholunate gap and a partial rotatory subluxation of the scaphoid, these patients experienced only mild or no pain and no carpal instability or deformity (3).

Here, we report the case of a monolateral non-traumatic SLD in a 10-year-old girl that led to an important range of motion impairment with no wrist pain. This case represents a rarity for no previous history of trauma, young age, and no pain.

## Case description

During the Covid lockdown, a 10-year-old female confessed to her parents that she was not able to perform some gym exercises with her sister because of left wrist extension loss. She did not feel any pain and she discounted recent or previous trauma. Her report was fairly reliable because she did not play any sport for a long time and had been spending a lot of time indoors since the beginning of the pandemic. None of her family members noticed her wrist condition before.

A month later, she was seen in our private practice.

Physical examination of patients with SLD usually reveals wrist edema, tenderness point at the dorsal SL interval, and pain during extension and radial deviation wrist movement. A palpable “clunk” can be felt during the performance of the Watson shift test (1), which involves placing an examiner thumb on the distal pole of the scaphoid on the palmar side and moving the wrist from the extended-ulnar deviation to the flexed-radial deviation with constant pressure: this noise indicates scapholunate instability.

In our patient, the range of motion in the wrist was impaired: she had complete flection (85°) but no extension (0°). She had no pain during passive or active mobilization and no edema.

Generally, she was a heathy patient with no underlining chronic disease in her medical history, no drug therapy and no past surgery.

**Figure 1:**
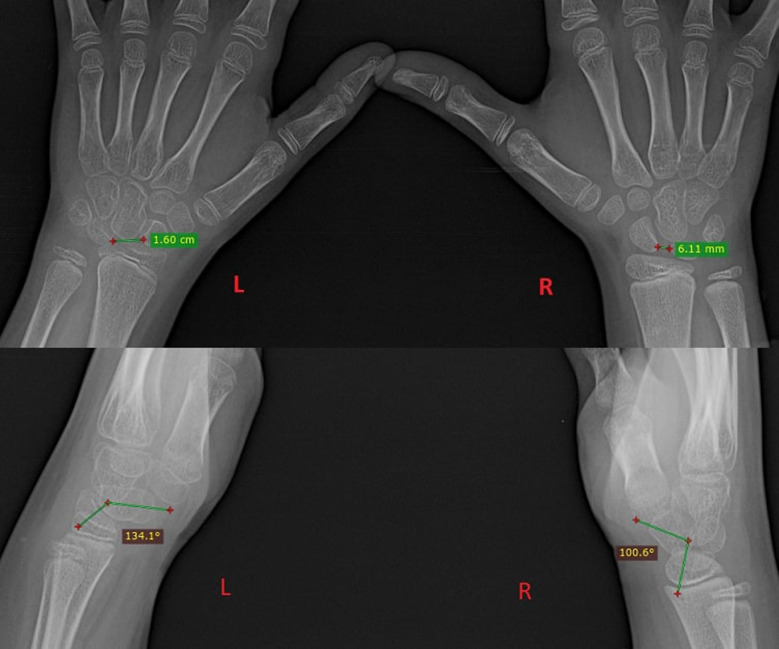
Presurgery X-ray: a comparison between an injured wrist (left) and a healthy wrist (right) in PA and lateral views; measuring the scapho-lunate distance and angle.

Her recent X-ray revealed a 16 mm scapholunate gap in the anteroposterior roentgenogram (the Terry Thomas sign) and a scapholunate angle of 134° on the lateral view (Figure 1).

## Diagnostic assessment

According to Cautilli and Wehbe, the normal distance between the scaphoid and the lunate in a posterior-to-anterior radiograph is 3 mm or less in an adult skeleton (4). In a child, this measurement is usually larger due to the presence of a growth nucleus (5), and it becomes necessary to compare the opposite wrist X-ray (Figure 1).

In a lateral radiograph, normally the radius and lunate should be perfectly aligned and the scapholunate angle ranges from 30° to 60°. In a scapholunate dissociation, in the lateral view, there is an abnormal dorsal slant of the lunate (DISI deformity) and a rotary subluxation of the scaphoid (6).

The “cortical ring” or “signet ring” sign consists of a radiopaque circle line visible on the posteroanterior hand X-ray in the case of SLD. It represents an overlapping view of the scaphoid tubercle on its axis when the scaphoid is rotated. Sometimes it could be normally present too, so the presence of this sign should be evaluated in light of clinical findings (1).

In our patient, MRI of her left wrist proved useful for definitive diagnosis, demonstrating SLD and a perilunate subluxation pattern. Acute trauma MRI also showed a ligamentous tear with a fluid presence between the lunate and the ligament (bright signal).

In view of the above findings, we also submitted her wrist to a 3D CT.

We did not do a diagnostic arthroscopy for some reasons: as we had already obtained the diagnosis with MRI and CT, we reasoned that the lesion needed an open surgery (direct reduction was necessary in inveterate bone dislocations); we also considered the fact that the radio-carpal space is small in adults and, smaller in children, so the risk of not having a good surgical view was high.

During our first examination of the girl, we noticed a generalized joint laxity in her body, with a Beighton score of 6/9 points. In this setting, like other authors (7), we suggested that congenital or developmental ligamentous laxity may be the SLD cause. Even if hyperlaxity was present and she always denied previous wrist trauma, we could not exclude without any doubt a traumatic cause of the disease: children sometimes do not dare to report an injury.

## Therapeutic intervention

SL acute dissociation needs urgent surgical intervention (within 6 weeks) to decrease the risk of carpal deformity, arthrosis, and severe wrist handicap. The recognition of early carpal instabilities is very important, in order to avoid non-diagnosed hand lesions that lead to chronic pain and long-term degenerative changes, including SL-advanced collapse, known as SLAC wrist (1).

Our patient underwent open surgery reconstruction using bone k-wire fixation and anchor suture. She received IV antibiotic therapy before and after her surgery.

**Figure 2:**
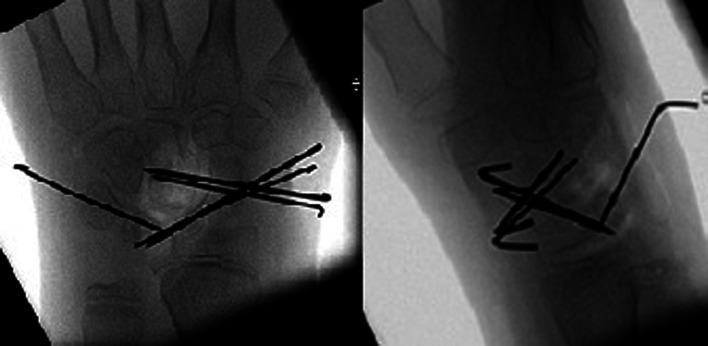
Postsurgery X-ray: carpal bones reduced and fixed with k-wires.

A dorsal skin incision on the left wrist was performed, and the radial sensitive nerve was isolated and protected. We partially opened the 4th extensor compartment. Berger-Bishop dorsal capsulotomy was performed to expose the carpal bones. Then, the periscaphoid synovial tissue was removed and the lesions were stabilized: the scapholunate and lunotriquetral ligaments were torn, and the capitate migrated in the scapholunate interspace. Under fluoroscopy, we reduced the dislocated bones and fixed them with percutaneous 1 mm k-wires (two wires to fix the scaphoid and lunate, two wires to fix the scaphoid and capitate, and one wire to fix the lunate and triquetrum). Finally, the scapholunate ligaments were repaired using bone-absorbable anchor sutures (Figure 2). After capsular, IV compartment, and skin suturing, the wrist was immobilized in a volar cast.

## Follow-up and outcomes

K-wires were removed after 8 weeks. At the first examination, the patient had a 20° active wrist flexion and 40° extension; she did not have any problems with her finger movement and there was good skin closure. She was referred to a hand therapist for passive and active exercises, 2 times a week, so that she could recover her wrist range of motion.

**Figure 3:**
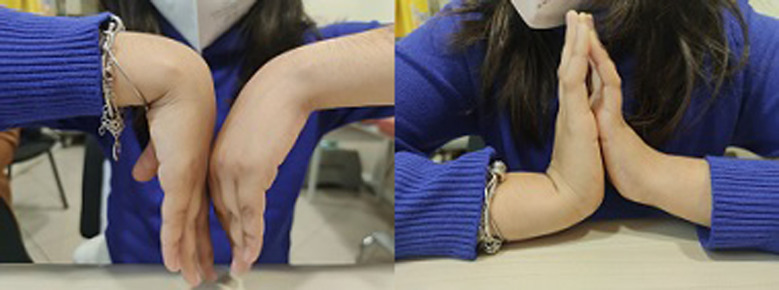
Wrist mobility 10 months after the performance of the surgery.

**Figure 4:**
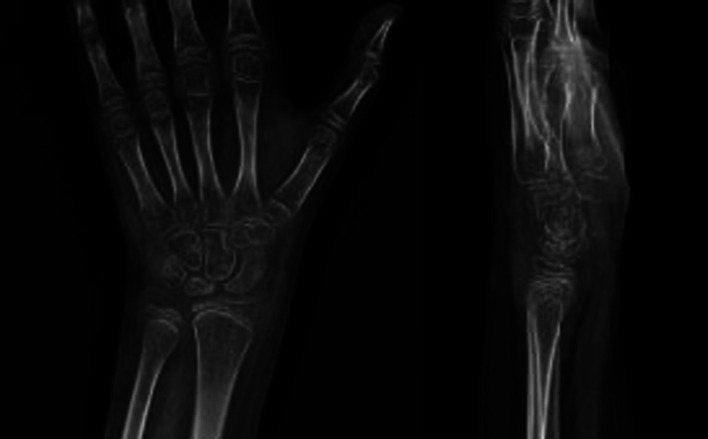
An injured wrist X-ray 10 months after the surgery.

The patient resumed her daily life activities (included sport) within 12 months. In her last clinical examination, 10 months after the surgery, she regained almost complete wrist extension (80°) and showed further improvement (50°) in flexion (Figure 3) and had no pain during the performance of exercises. X-ray showed a restored carpal alignment (Figure 4).

## Discussion

Vance et al. reported a case of asymptomatic bilateral SLD in an adult and stated that ligamentous laxity could be a causative factor for this condition (6). We could hypothesize that it was etiopathogenesis in our case.

SLD and perilunate dislocations are rare in adults, but even rarer in children. To the best of our knowledge, only eight cases of perilunate dislocation in the pediatric population have been reported in the literature, and it was only in our case, there was no previous history of trauma.

Moreover, we noticed that in every case that was reported, the age range of patients was between 9 and 12 years, and most of them had an additional injury (different radius fractures).

Multiple surgical techniques have been proposed to treat this problem. For acute SLD, currently, most authors recommended either a closed or open reduction with temporary internal fixation. The treatment of chronic SLD is more controversial than others and is fraught with more risks. Also, it is a much-discussed topic. During the subacute window, between 3 and 8 weeks after injury, the ligaments do not heal spontaneously and the poor quality of tissue does not allow repair by direct suture (8). Tendon grafts have been reported to replace the scapholunate ligament, but this procedure is technically demanding. During one's childhood, an intraosseous reconstruction using the flexor carpi radialis tendon tunneled through the scaphoid should be dismissed for growth plate presence in bones obviously. An extraosseous reconstruction using the dorsal capsule Brunelli flap and dorsal radiocarpal ligament has been described (9).

Non-surgical techniques, such as simply cast immobilization or closed reduction, are unsuitable for people with dislocated bones, with the possibility of such patients developing arthritis being high. Early diagnosis and treatment of acute scapholunate injury has been reported to be the most effective way of preventing degenerative lesions (8).

Management of acute carpal bone dislocations includes early closed reduction and percutaneous wire fixation for 8 weeks. However, in chronic injury, and if closed reduction fails, open reduction techniques have been described with good outcomes (10).

## Conclusion

In summary, although carpal lesions are rare in children, we highlight the importance of a meticulous physical examination and radiological analysis for finding carpal instability injuries. If missed, they can cause devastating outcomes in wrist function.

Treatment for such injuries in the young age group is beset with risks and is controversial.

Therefore, we encourage more of our colleagues to flush out these carpal lesions and report about them in order to improve management outcomes in the future.

## Patient perspective

Our case demonstrates that the patient has had a good clinical outcome. Her physio-rehabilitation program has been one of long duration. Twelve months postoperatively, the partial range of motion of her injured wrist was evident, but we see more room for improvement. Most studies have shown excellent clinical results after an average of 2.4 years (11). Therefore, we will continue to follow up this case.

## Data Availability

The raw data supporting the conclusions of this article will be made available by the authors without undue reservation.
